# Hypoxia Induces Mitochondrial Defect That Promotes T Cell Exhaustion in Tumor Microenvironment Through MYC-Regulated Pathways

**DOI:** 10.3389/fimmu.2020.01906

**Published:** 2020-08-21

**Authors:** Yi-Na Liu, Jie-Feng Yang, Dai-Jia Huang, Huan-He Ni, Chuan-Xia Zhang, Lin Zhang, Jia He, Jia-Mei Gu, Hong-Xia Chen, Hai-Qiang Mai, Qiu-Yan Chen, Xiao-Shi Zhang, Song Gao, Jiang Li

**Affiliations:** ^1^Collaborative Innovation Center for Cancer Medicine, State Key Laboratory of Oncology in South China, Guangzhou, China; ^2^Key Laboratory of Gene Engineering of the Ministry of Education, State Key Laboratory of Biocontrol, School of Life Sciences, Sun Yat-sen University, Guangzhou, China; ^3^Department of Biotherapy, Sun Yat-sen University Cancer Center, Guangzhou, China; ^4^Department of Nasopharyngeal Carcinoma, Sun Yat-sen University Cancer Center, Guangzhou, China; ^5^Guangzhou Regenerative Medicine and Health Guangdong Laboratory, Guangzhou, China; ^6^Department of Research and Development, Shenzhen Institute for Innovation and Translational Medicine, Shenzhen International Biological Valley-Life Science Industrial Park, Shenzhen, China

**Keywords:** T cell exhaustion, mitochondrial dynamics, miR-24, nasopharyngeal carcinoma, MYC

## Abstract

T cell exhaustion is an obstacle to immunotherapy for solid tumors. An understanding of the mechanism by which T cells develop this phenotype in solid tumors is needed. Here, hypoxia, a feature of the tumor microenvironment, causes T cell exhaustion (T_Exh_) by inducing a mitochondrial defect. Upon exposure to hypoxia, activated T cells with a T_Exh_ phenotype are characterized by mitochondrial fragmentation, decreased ATP production, and decreased mitochondrial oxidative phosphorylation activity. The T_Exh_ phenotype is correlated with the downregulation of the mitochondrial fusion protein mitofusin 1 (MFN1) and upregulation of miR-24. Overexpression of miR-24 alters the transcription of many metabolism-related genes including its target genes *MYC* and fibroblast growth factor 11 (*FGF11*). Downregulation of *MYC* and *FGF11* induces T_Exh_ differentiation, reduced ATP production and a loss of the mitochondrial mass in T cell receptor (TCR)-stimulated T cells. In addition, we determined that MYC regulates the transcription of *FGF11* and *MFN1*. In nasopharyngeal carcinoma (NPC) tissues, the T cells exhibit an increased frequency of exhaustion and loss of mitochondrial mass. In addition, inhibition of miR-24 signaling decreases NPC xenograft growth in nude mice. Our findings reveal a mechanism for T cell exhaustion in the tumor environment and provide potential strategies that target mitochondrial metabolism for cancer immunotherapy.

## Introduction

T cell exhaustion, which is characterized by progressive decreases in proliferation, cytokine production, and cytotoxic capabilities, is an acquired state of T cell dysfunction and a hallmark of cancer and chronic viral infections (1). During chronic viral infections, high antigen loads induce the T cell exhaustion (T_Exh_) phenotype (2). However, the mechanism by which T cells develop an exhaustion phenotype in human cancers is not completely understood, although the tumor microenvironment (TME) and intrinsic programmed cell death protein 1 (PD-1) and extrinsic immunoregulatory cytokine negative regulatory pathways are considered important factors (3). Immunotherapies based on checkpoint inhibition or chimeric antigen receptor (CAR) T cells that aim to reverse T_Exh_ in cancers by targeting negative regulatory pathways have been strikingly effective (4, 5). Nevertheless, the majority of patients, particularly those suffering from solid tumors, fail to benefit from these therapies. This failure is partly due to the adverse effect of the TME on the function of tumor-infiltrating lymphocytes (TILs) (6, 7). Thus, identification of the mechanisms underlying the development of the TME-induced T_Exh_ phenotype in solid tumors is necessary.

Hypoxia is a prominent feature of the TME in solid tumors and is considered a major factor driving adaptation toward host immunosurveillance evasion (8, 9). Although the exact mechanism remains unclear, hypoxic stress is thought to impede immune cell functions by inducing the expression and production of immunosuppressive molecules and metabolites in tumor cells (10, 11). We previously reported that hypoxia induces the enrichment of miR-24 in nasopharyngeal carcinoma (NPC) cells and NPC-derived exosomes and thereby disrupts the activity of T cells (12). In tumor cells, hypoxia triggers angiogenesis, the epithelial–mesenchymal transition (EMT) and metabolic reprogramming in addition to immune modulation (9, 13, 14). However, the direct response of human TILs to hypoxic stress and the relationship between hypoxia and T_Exh_ remain to be elucidated.

T cell fate is closely related to metabolic conditions. T cell activation occurs concomitantly with the engagement of aerobic glycolysis and induces increases in mitochondrial oxidative phosphorylation (OXPHOS) activity (15). Competition with cancer cells in the TME leads to metabolic dysregulation of TILs, which is a hallmark of the T_Exh_ phenotype and is often coupled with mitochondrial deficiencies (16, 17). T cells rapidly lose mitochondrial mass upon entering malignant tissues. In most eukaryotic cells, mitochondria undergo regulated and frequent fusion and fission processes. Fusion contributes to the formation of a healthy mitochondrial network and promotes OXPHOS activity. It is unclear whether impairment of mitochondrial dynamics, as reflected by imbalance between fusion and fission processes, is involved in the development of the T_Exh_ phenotype (18). The dynamin-like GTPases mitofusin 1 and mitofusin 2 (MFN1 and MFN2, respectively) initiate mitochondrial fusion by catalyzing the tethering and merging of outer membranes. Depletion of MFN1/2 leads to mitochondrial fragmentation and ultimately to mitochondrial dysfunction (19–21). In this study, we show that impairment of mitochondrial dynamics is closely associated with the exhaustion of both hypoxia-treated T cells and patient-derived TILs and that the exhaustion phenotype is induced through the miR-24-MYC-MFN1 axis. These data establish a new pathway for the direct induction of the T_Exh_ phenotype by hypoxia through the regulation of mitochondrial dynamics and provide novel strategies for the development of effective immunotherapeutic approaches.

## Materials and Methods

### Sample Collection, T Cell Culture, and Exosome Isolation

Fresh tumor specimens and peripheral blood samples were collected from 10 NPC patients at the time of their first diagnosis at Sun Yat-sen University (SYSU) Cancer Center, Guangzhou, China, in 2015. The detailed clinical data are summarized in Supplementary Table 1. Ten age-matched healthy donors (HDs) were included in this study. This study was conducted in accordance with the Declaration of Helsinki. All the patients and HDs provided written consent. The study was approved by the Research Ethics Committee of SYSU Cancer Center (GZR2013-040).

For isolation of TILs, biopsied NPC tissues were minced into small pieces and digested with collagenase type IV (0.1 μg/ml) for 2 h. The protocol for TIL culture is described in detail in Supplemental Experimental Procedures.

### Reagents and Antibodies

Detailed information about the antibodies used in this study is provided in Supplementary Table 2.

### Plasmid Construction, Lentivirus Production and Transduction

miR-24 (HmiR0146-MR03), scrambled control (CmiR0001-MR03), miR-24 sponge (HmiR-AN0349-AM03), scrambled sponge control (CmiR-AN0001-AM03), scrambled control short hairpin RNA (shRNA) (shControl), MYC-specific shRNA (shMYC), and FGF11-specific shRNA (shFGF11, HSH067396-3-LVRU6MP) sequences were separately cloned into lentivectors (GeneCopoeia). This protocol is described in detail in Supplemental Experimental Procedures.

### T Cell Proliferation and Differentiation Assay

This protocol is described in detail in Supplemental Experimental Procedures.

### RNA Sequencing (RNA-Seq) and Analysis

This protocol is described in detail in Supplemental Experimental Procedures. The RNA-seq data have been deposited in the NCBI Gene Expression Omnibus (GEO) under the Accession Code GSE110523.

### Real-Time RT-qPCR and Immunoblotting

This protocol is described in detail in Supplemental Experimental Procedures.

### Determination of Cell Viability

A CellTiter-Glo® Luminescent Cell Viability assay was used to determine the number of viable cells in culture based on the quantification of ATP produced in the cell culture supernatant after 48, 72, and 96 h under different treatment conditions. CellTiter-Glo® reagent (100 μl) and cell culture supernatant (100 μl) were added to each well of a 96-well plate, mixed for 2 min on an orbital shaker, and incubated at room temperature for 10 min to stabilize the luminescent signal. Cell luminescence was detected using a Tecan Spark™ 10 M microplate reader.

### Metabolism Measurements

Twenty-four-well XF cell culture microplates were coated with 0.1 mg/ml poly-D-lysine (Sigma-Aldrich) and then washed with PBS (phosphate-buffered saline). The extracellular acidification rate (ECAR) and oxygen consumption rate (OCR) were detected using a 24-well XF or XFe extracellular flux analyzer. For the ECAR assays, the cells (2 × 10^5^) were plated in assay medium (XF Base Medium containing 2 mM L-glutamine) supplemented with 10 mM glucose, 1 μM oligomycin (Seahorse Bioscience), and 50 mM 2-deoxy-D-glucose (2-DG) (Sigma-Aldrich) under basal conditions. For the OCR assays, cells (2 × 10^5^) were plated in assay medium (XF Base Medium containing 10 mM glucose, 2 mM L-glutamine, and 1 mM sodium pyruvate) under basal conditions and stimulated with 1 μM oligomycin, 1.0 μM fluoro-carbonyl cyanide phenylhydrazone (FCCP), and 0.5 μM rotenone/antimycin A according to the manufacturer's instructions and our previous study (22).

### Mitochondrial Staining and Microscopic Observation

This protocol is described in detail in Supplemental Experimental Procedures.

### Electron Microscopy

Tumor tissues from NPC patients were fixed, sectioned, stained, and coated at the SYSU Electron Microscopy core facility. The images were visualized using FEI Tecnai transmission electron microscopes.

### Luciferase Reporter Assay

This protocol is described in detail in Supplemental Experimental Procedures.

### Xenograft Mouse Model

All animal experiments were performed in accordance with protocols approved by the Institutional Animal Care and Use Committee of SYSU, Guangzhou, China (L102012018060P). The *in vivo* experiments were performed using 4-week-old female nude athymic mice (BALB/c-nu/nu, Harlan). Briefly, 2 × 10^5^ CNE2 cells resuspended in 100 μl of PBS were injected intravenously into the tail vein. After 1 week of *in vitro* pretreatment under different conditions for 1 week, TILs (4 × 10^5^ and 1.2 × 10^6^ cells) from NPC patients were injected intravenously after tumor challenge and every 2 weeks thereafter. The *in vivo* treatment conditions for the TILs are described below. First, 1 × 10^6^ TILs were plated in an anti-CD3 antibody (OKT3)-coated 24-well plate and transfected with lenti-sponge-control (group 2 [G2]), lenti-miR-24-sponge (group 3 [G3]), lenti-shMYC (group 4 [G4]), or lenti-shMYC + 10 μM Mdivi-1 (a mitochondrial fission inhibitor) + 25 μM bezafibrate (group 5 [G5]) for three days. A xenograft + PBS group (group 1 [G1]) was included as a control. The cells were then harvested for injection into the mice. The mice were sacrificed 3 weeks after the last treatment. Their lungs were removed and weighed, and tumor nodes visible to the naked eye were counted. For pathological examination, the lungs were fixed with formalin, embedded in paraffin, sectioned consecutively at a thickness of 4 μm, and stained with hematoxylin and eosin (H&E). The tumor nodes in each field were counted under a microscope at 10x magnification. All mouse experiments were performed with groups of five to six mice (the exact numbers are specified in the figure legends). The mice were randomly grouped into the treatment or corresponding control groups, and the operators were blinded to the group assignments.

### Statistical Analysis

This protocol is described in detail in Supplemental Experimental Procedures.

## Results

### Hypoxia Induces the T_Exh_ Phenotype and Alters Mitochondrial Metabolism and Dynamics in T Cells

**Figure 1:**
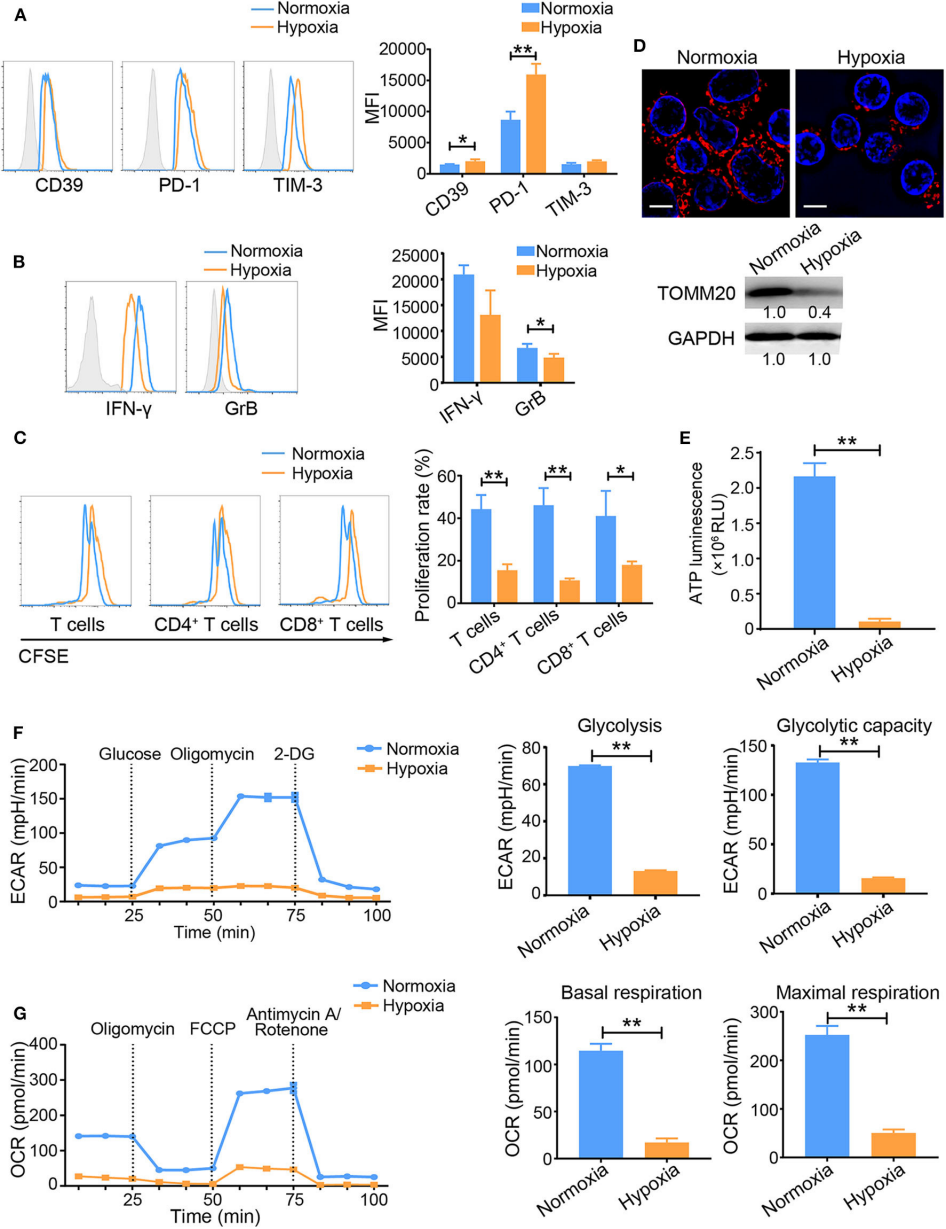
Hypoxia induces the T_Exh_ phenotype and energy metabolism dysregulation in activated T cells. T Cells were isolated from the peripheral blood of healthy donors, plated in the wells of OKT3-coated 24-well plates, and treated under the different indicated conditions. **(A,B)** Graphs showing the expression levels of inhibitory molecules, including CD39, PD-1 and TIM-3 **(A)**, and the effector cytokines IFN-γ and GrB **(B)** in activated T cells exposed to normoxic and hypoxic conditions for 48 h. **(C)** Proliferation of activated T cells exposed to normoxic and hypoxic conditions for 48 h. **(D)** Representative structured illumination microscopy images of activated T cells cultured under normoxic or hypoxic conditions for 48 h and immunoblot showing the levels of TOMM20. Images from one of three experiments are shown. The mitochondria are red (MitoTracker Deep Red), and the nuclei are blue (DAPI). Scale bar, 50 μm. **(E–G)** Analysis of energy metabolism parameters, including ATP production **(E)**, ECAR (glycolysis and glycolytic capacity) **(F)** and OCR (basal and maximal respiration) **(G)** in OKT3-stimulated T Cells exposed to normoxic or hypoxic conditions for 48 h; the values were normalized to the number of cells. All the data were obtained from at least three independent experiments. The data are presented as the means ± SEMs. The error bars represent the SEMs. *P < 0.05, **P < 0.01 (two-tailed Student's *t*-test).

Hypoxia subverts the immune system and promotes tumorigenesis (23, 24). However, the direct effects of hypoxia on tumor-infiltrated T cells have not been fully elucidated. To explore this issue, we first investigated the differences in activated T cells under normoxic *vs*. hypoxic conditions *in vitro*. T cells were activated with a well-established system in which an anti-CD3 antibody (OKT3) was used to mimic the human TCR-mediated signal. Compared with normoxia-treated cells, activated T cells exposed to hypoxia for 48 h showed increased expression of hypoxia-inducible factor 1 alpha (HIF-1α) (Supplementary Figures 1A,B), CD39, PD-1, and TIM-3, but decreased secretion of the effector cytokines interferon-gamma (IFN-γ) and granzyme B (GrB) (Figures 1A,B). Importantly, hypoxia suppressed the proliferation of activated T cells, including both CD4^+^ and CD8^+^ T cells (Figure 1C). Accordingly, fewer mitochondria and decreased TOMM20 (translocase of outer mitochondrial membrane 20 homolog) expression were observed in OKT3-activated T cells exposed to hypoxic stress than in T cells exposed to normoxic condition (Figure 1D). Hypoxia also reduced total ATP production by activated T cells (Figure 1E). To understand the metabolic properties of these activated T cells, we measured their ECARs and OCRs as indicators of aerobic glycolysis and OXPHOS activity, respectively. Both the ECAR and OCR values were substantially lower in T cells under hypoxia than in T cells under normoxia. (Figures 1F,G). Under hypoxic conditions, the expression of glycolysis-related enzymes was markedly increased (Supplementary Figure 1C). These data suggest that hypoxia induces the T_Exh_ phenotype and mitochondrial dysfunction.

### Inhibition of MFN1-Mediated Mitochondrial Fusion Induces T_Exh_

**Figure 2:**
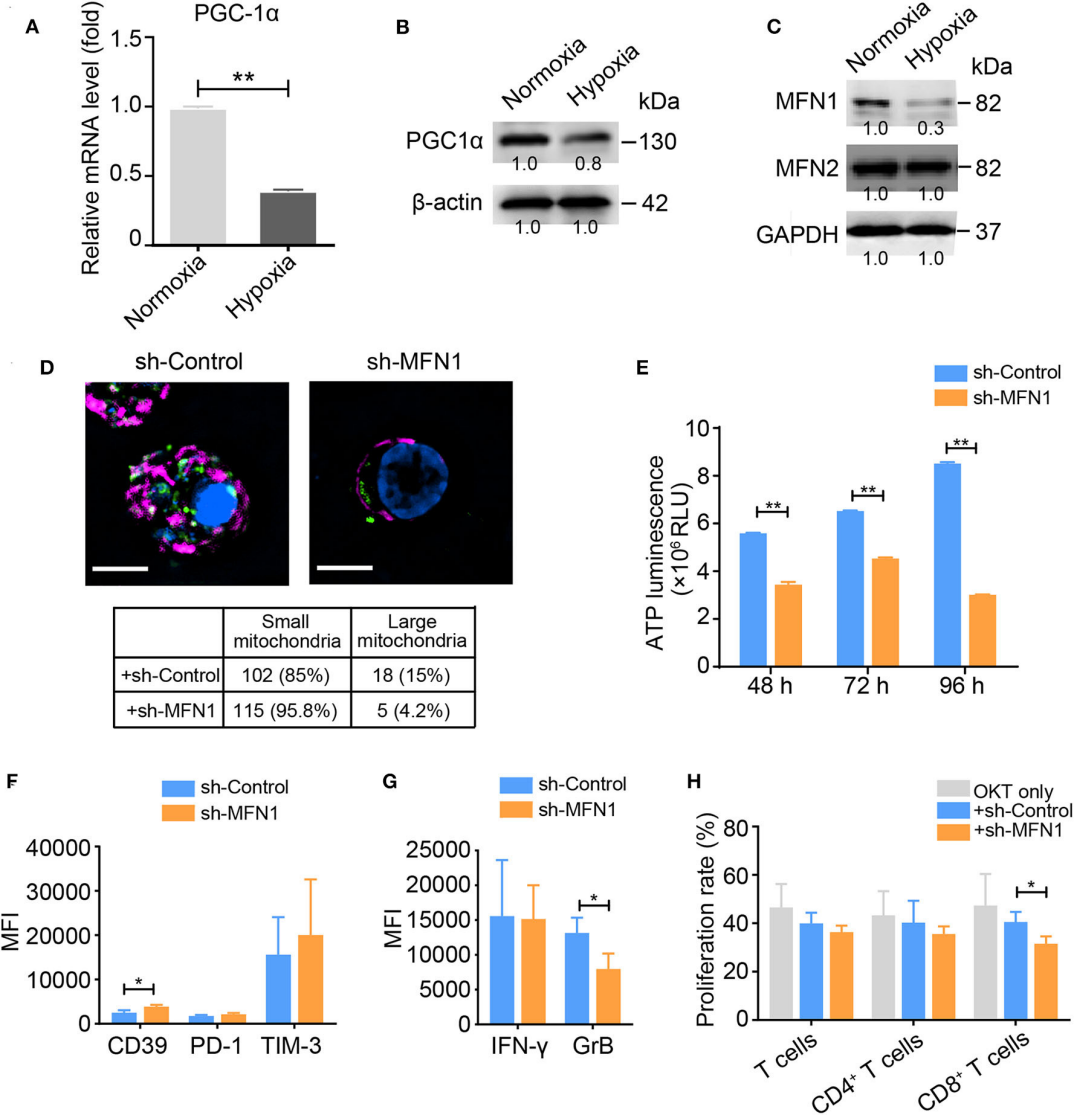
MFN1 is essential for hypoxia-induced T_Exh_ and mitochondrial energy metabolic reprogramming. **(A,B)** Levels of the PGC-1α mRNA and protein in activated T cells cultured under normoxic and hypoxic conditions. **(C)** Immunoblot analysis of activated T cells cultured under normoxic or hypoxic conditions for 48 h using the indicated antibodies; the data from one of three independent experiments are shown. **(D)** Representative structured illumination microscopy images of activated T cells cultured under the indicated treatment conditions for 48 h; the images from one of three experiments are shown. The mitochondria are shown in red (MitoTracker Deep Red), shControl and shMFN1 are shown in green (GFP), and the nuclei are shown in blue (DAPI). Scale bar, 50 μm. The small and large mitochondria that formed per microscopic field were counted. **(E)** ATP production in shMFN1 and shControl vector-treated T cells was measured at 48, 72, and 96 h. **(F,G)** Expression levels of inhibitory molecules, including CD39, PD-1, and TIM-3 **(F)**, and the effector cytokines IFN-γ and GrB **(G)** in activated T cells treated with the lenti-shMFN1 or lenti-control vector. **(H)** Proliferation of activated T cells transduced with the lenti-shMFN1 or lenti-control vector. All data were obtained from at least three independent experiments. The error bars represent the SEMs. *P < 0.05, **P < 0.01 (two-tailed Student's *t*-test).

Because we observed a prominent loss of mitochondrial mass in hypoxia-treated activated T cells, we examined the expression profile of peroxisome proliferator-activated receptor gamma coactivator 1-alpha (PGC1α), a transcription factor involved in mitochondrial biogenesis. Under hypoxic conditions, the activated T cells displayed a decreased PGC1α expression at both the mRNA and protein levels (Figures 2A,B), suggesting that PGC1α might account for the mitochondrial loss observed in hypoxia-treated T cells. Intriguingly, we discovered that the expression level of the mitochondrial fusion protein MFN1 was significantly decreased in activated T cells under hypoxia, while the level of MFN2 was also slightly decreased (Figure 2C). Compared with control T cells, OKT3-activated T cells in which endogenous MFN1 was knocked down presented small and fragmented mitochondria that were dispersed in the cytoplasm (Figure 2D), as well as decreased ATP production, which was mainly due to the suppression mitochondrial OXPHOS (Figure 2E). MFN1 knockdown in OKT3-activated T cells increased the expression of CD39, decreased the secretion of IFN-γ and GrB, and resulted in poor proliferation (Figures 2F–H). These data suggest that mitochondrial fusion deficiency caused by MFN1 downregulation in activated T cells is closely related to the T_Exh_ phenotype.

### Hypoxia-Induced MiR-24 Expression Promotes the Progression of Activated Human T Cells to the Exhausted Phenotype by Regulating Mitochondrial Dynamics

**Figure 3:**
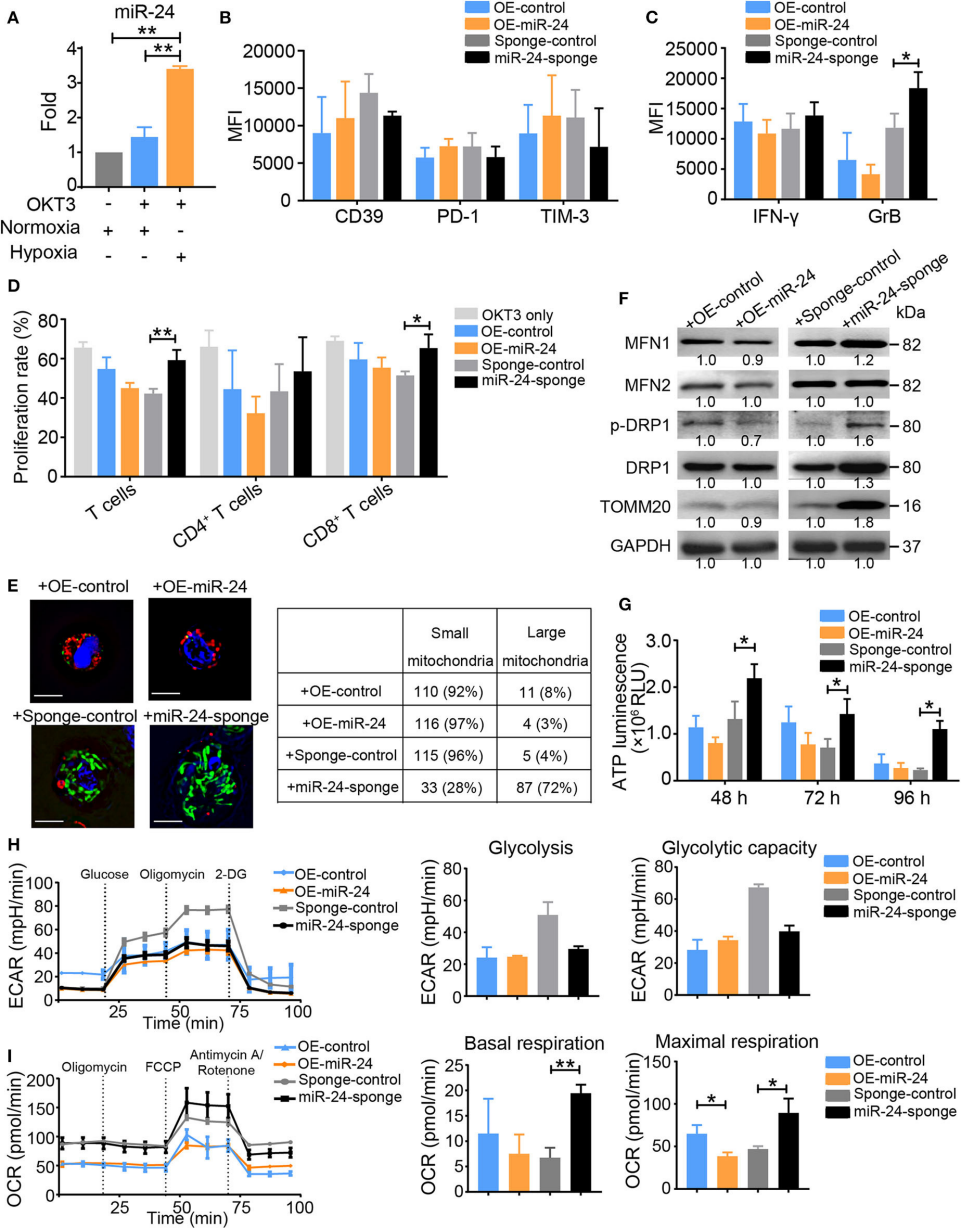
Ectopic expression of miR-24 induces T_Exh_ *in vitro*. **(A)** Real-time RT-qPCR assay showing the relative miR-24 levels in OKT3-activated T cells cultured under normoxic and hypoxic conditions; T cells that were not stimulated with OKT3 were included as controls. **(B,C)** Graphs showing the expression levels of inhibitory molecules, including CD39, PD-1 and TIM-3 **(B)**, and the effector cytokines IFN-γ and GrB **(C)** in activated T cells transduced with the lenti-miR-24 (OE-miR-24), lenti-miR-24-sponge (miR-24-sponge), or corresponding lenti-control vector, as determined using flow cytometry. **(D)** Proliferation of activated T cells treated with the lenti-miR-24, lenti-miR-24-sponge, or corresponding lenti-control vector for 3 days. **(E)** Representative structured illumination microscopy images of activated T cells cultured under the indicated treatment conditions for 48 h; images from one of three experiments are shown. The mitochondria are shown in green or red (MitoTracker Green or MitoTracker Deep Red, respectively), OE-control and OE-miR-24 are shown in green (GFP), sponge-control and miR-24-sponge are shown in red (m-Cherry), and the nuclei are shown in blue (DAPI). Scale bar, 50 μm. The small and large mitochondria that formed per microscopic field were counted. **(F)** Immunoblot analysis of activated T cells after the indicated treatments for 72 h *in vitro*. Representative data from one of three independent experiments are shown. **(G)** ATP production in activated T cells treated with the lenti-miR-24, lenti-miR-24-sponge or lenti-control vector was measured at 48, 72, and 96 h. **(H,I)** The ECAR and OCR values in activated T Cells were measured 48 h after transduction with the lenti-sponge-control, lenti-miR-24-sponge, lenti-OE-control, and lenti-OE-miR-24; the values were normalized to the number of cells. All data were obtained from at least three independent experiments. The data are presented as the means ± SEMs. The error bars represent the SEMs. *P < 0.05, **P < 0.01 (one-way ANOVA and two-tailed Student's *t*-test).

In a previous study, we found that hypoxia induces exosomal miR-24 secretion in NPC and that this process is linked to T cell dysfunction (12). In the present study, miR-24 expression was also increased in activated T cells under hypoxia (Figure 3A). OKT3-activated T cells overexpressing miR-24 (OE-miR-24) exhibited increased levels of CD39, PD-1, and TIM-3, but decreased levels of IFN-γ and GrB and poor proliferation, while the ablation of endogenous miR-24 (miR-24-sponge) produced the opposite effect on the expression of these inhibitory molecules and the secretion of effector cytokines (Figures 3B–D). Based on these data, overexpression of miR-24 in T cells induces the T_Exh_ phenotype *in vitro*. Moreover, the NPC cell-derived exosomes, which exhibit miR-24 enrichment (12), also induced the T_Exh_ phenotype *in vitro* (Supplementary Figures 2A,B).

OKT3-activated T cells in which endogenous miR-24 was knocked down contained densely packed, moderately tubulated mitochondria, whereas activated T cells overexpressing miR-24 showed relatively small and fragmented mitochondria (Figure 3E). The levels of MFN1, MFN2, p-DRP1 (phospho-dynamin related protein 1) and TOMM20 were increased in miR-24 sponge-treated T cells, but decreased in miR-24-treated T cells (Figure 3F). The ATP concentrations in the supernatants of activated T cell cultures were notably increased at multiple time points (48, 72, and 96 h) after endogenous miR-24 was knocked down, but were decreased upon miR-24 overexpression (Figure 3G), suggesting that miR-24 reduces ATP production in activated T cells. Activation of T cells is believed to elevate both aerobic glycolysis and mitochondrial OXPHOS activity to allow more robust ATP production (24). Although the ECAR and basal total oxygen consumption did not show marked differences between the miR-24-treated and miR-24 sponge-treated T Cells, the miR-24 sponge vector or /tumor cell line-derived exosome (T-EXO)-treated T Cells exhibited increases in the basal respiration and notable increases in the maximal respiration values (Figures 3H,I and Supplementary Figures 2C,D). These results indicate that miR-24 is a negative regulator of mitochondrial function.

### Transcriptome Analysis Identifies MYC as a Key Modulator of the MiR-24-Mediated Induction of the T_Exh_ Phenotype

**Figure 4:**
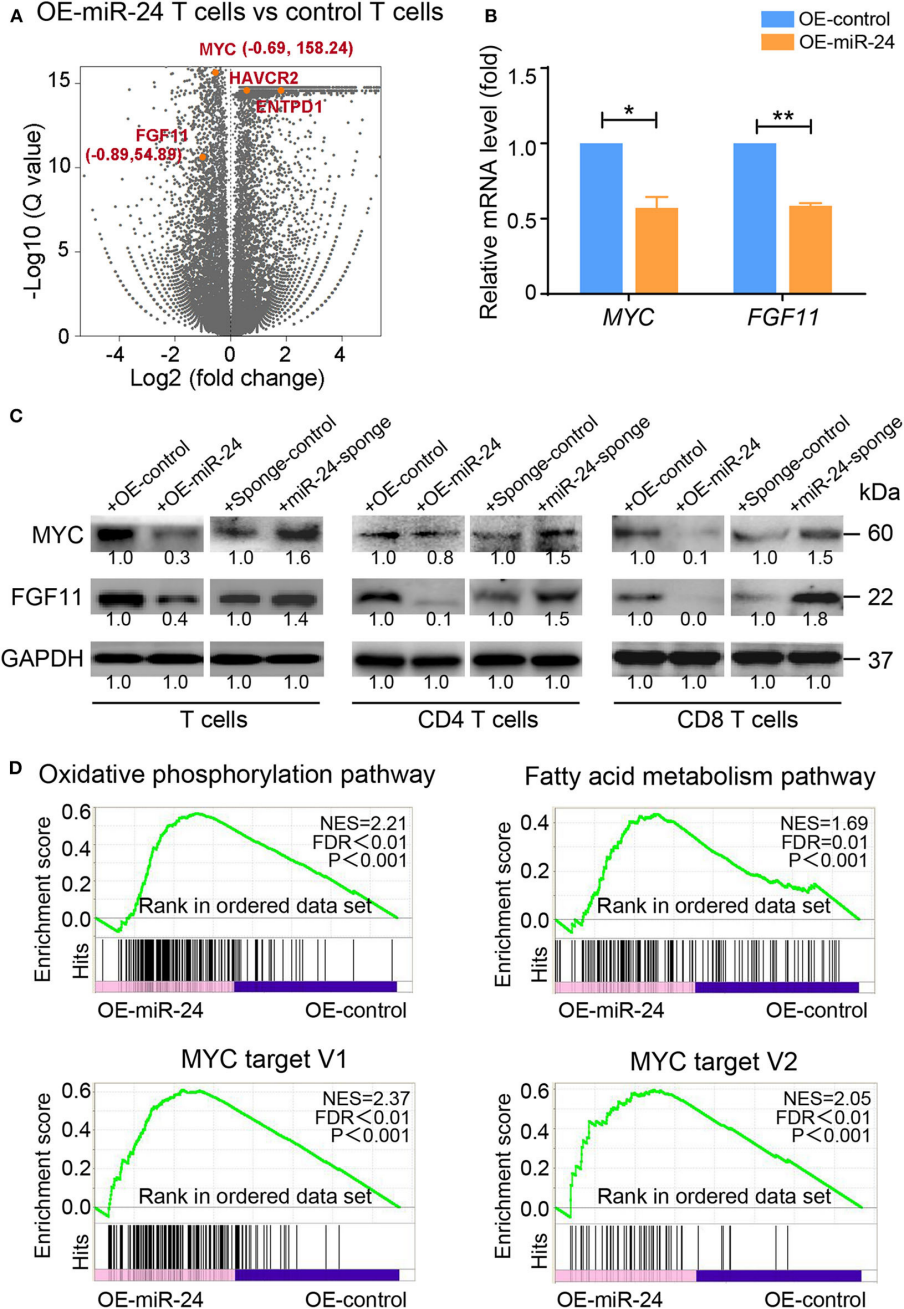
Exogenous miR-24 alters gene expression associated with cellular and metabolic processes in T cells. RNA-seq was performed on activated T cells transfected with control and miR-24-expressing lentiviruses *in vitro*. **(A)** Volcano plot showing the differential expression of all genes (gray) or the indicated miR-24 target genes *MYC* and *FGF11* and the exhaustion-related genes *ENTPD1* and *HAVCR2* (orange) in control vs. miR-24-expressing T cells. **(B,C)** The mRNA and protein levels of the miR-24 target genes MYC and FGF11 in activated T cells, including CD4^+^ and CD8^+^ T cells, transduced with the lenti-miR-24, lenti-miR-24-sponge or corresponding lenti-control vector were measured using real-time RT-qPCR and immunoblotting, respectively. **(D)** The gene set enrichment analysis (GSEA) revealed an enrichment of genes involved in the OXPHOS pathway, the fatty acid metabolism pathway and MYC target genes in control cells compared with miR-24-expressing T cells. NES, normalized enrichment score. All data were obtained from at least three independent experiments. *P < 0.05, **P < 0.01 (two-tailed Student's *t*-test).

To further investigate the detailed molecular mechanism responsible for the miR-24-mediated induction of the T_Exh_ phenotype at the posttranscriptional level, we investigated the changes in the expression profiles of activated T cells overexpressing miR-24 by RNA-seq. Among the differentially expressed genes observed in miR-24-treated *vs*. control T cells, several reported direct targets of miR-24, including *MYC* and *FGF11*, and genes encoding T cell-inhibitory ligands, such as *ENTPD1* and *HAVCR2*, were significantly altered (Figure 4A). We verified that the mRNA and protein levels of MYC and FGF11 in T cells, including CD4^+^ and CD8^+^ T cells, were suppressed by forced miR-24 overexpression and upregulated after miR-24 knockdown (Figures 4B,C). A pathway analysis revealed that the transcripts of genes involved in OXPHOS and fatty acid metabolism and MYC targets were significantly altered by miR-24 overexpression compared with the control condition (Figure 4D).

### MYC and FGF11 Are Essential for the Reprogramming of Mitochondrial Dynamics in T Cells

**Figure 5:**
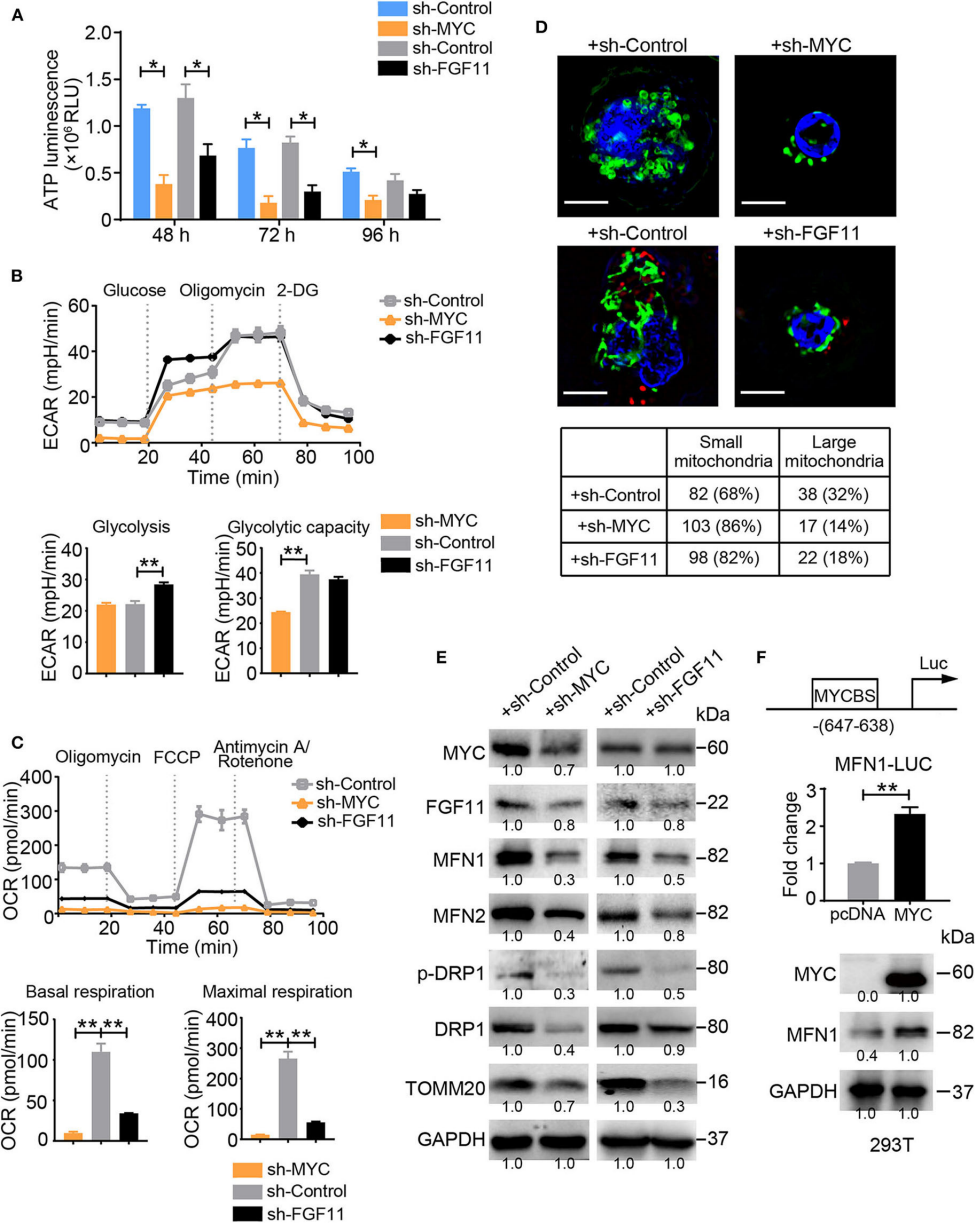
MYC and FGF11 are essential for mitochondrial energy metabolism reprogramming. **(A)** ATP production in shMYC, shFGF11 and shControl vector-transfected T cells was measured. **(B,C)** ECAR and OCR values of activated T Cells transfected with the shControl, shMYC, or shFGF11 vector; the values were normalized to the number of cells. **(D)** Representative structured illumination microscopy images of activated cells transfected with the shMYC, shFGF11, or shControl vector; images from one of three independent experiments are shown. The mitochondria are shown in green (MitoTracker Green), shControl and shFGF11 are shown in red (m-Cherry), and the nuclei are shown in blue (DAPI). Scale bar, 50 μm. The small and large mitochondria per field were counted under a microscope. **(E)** Immunoblot analysis of activated T cells transfected with the shMYC, shFGF11 or shControl vector using the indicated antibodies; the results from one of three independent experiments are shown. **(F)** Schematic showing the MYC-binding site (MYCBS) in the MFN1 promoter and the results of the luciferase reporter assay of the transcriptional regulation of MFN1 in 293T cells. All data were obtained from at least three independent experiments. The error bars represent the SEMs. *P < 0.05, **P < 0.01 (two-tailed Student's *t*-test).

According to a bioinformatic analysis (DIANA mirPath 2.0 software) and previous studies, MYC is a driving factor in the mechanism regulating cell metabolism and a direct target of miR-24, similar to FGF11 (25). We investigated whether MYC or FGF11 was involved in the regulation of glycolytic and mitochondrial energy respiration in activated T cells. Blockade of MYC or FGF11 significantly decreased the total ATP production in activated T cells at 48, 72, and 96 h (Figure 5A). The shFGF11- and shMYC-treated T Cells showed significantly increased basal or maximal ECAR values, respectively; meanwhile, both shMYC- and shFGF11-treated T cells exhibited significantly decreased basal and maximal respiration values (Figures 5B,C). Moreover, the mitochondrial size was substantially reduced in the shMYC- and shFGF11-treated activated T cells (Figure 5D), and the expression levels of MFN1/2, p-DRP1, DRP1, and TOMM20 were decreased in the shMYC- and shFGF11-treated T cells compared with the corresponding control T cells (Figure 5E). Although this result reflects a loss of mitochondrial mass, the change in mitochondrial morphology implies that the mitochondria in these cells also experienced inadequate fusion. We next sought to understand whether the canonical translational regulator MYC is involved in the regulation of mitochondrial fusion and to elucidate the underlying mechanism. We identified a MYC-binding site in the MFN1 promoter at a region spanning nucleotides −647 to −638 relative to the open reading frame and confirmed that MYC was able to induce the expression of the *MFN1* gene containing a corresponding sequence by performing a luciferase assay (Figure 5F). These observations indicate that MYC enhances mitochondrial OXPHOS activity and is closely related to mitochondrial fusion via MFN1.

### FGF11 Is a Signal Transducer Between MYC and MFN1 in the T_Exh_ Induction

**Figure 6:**
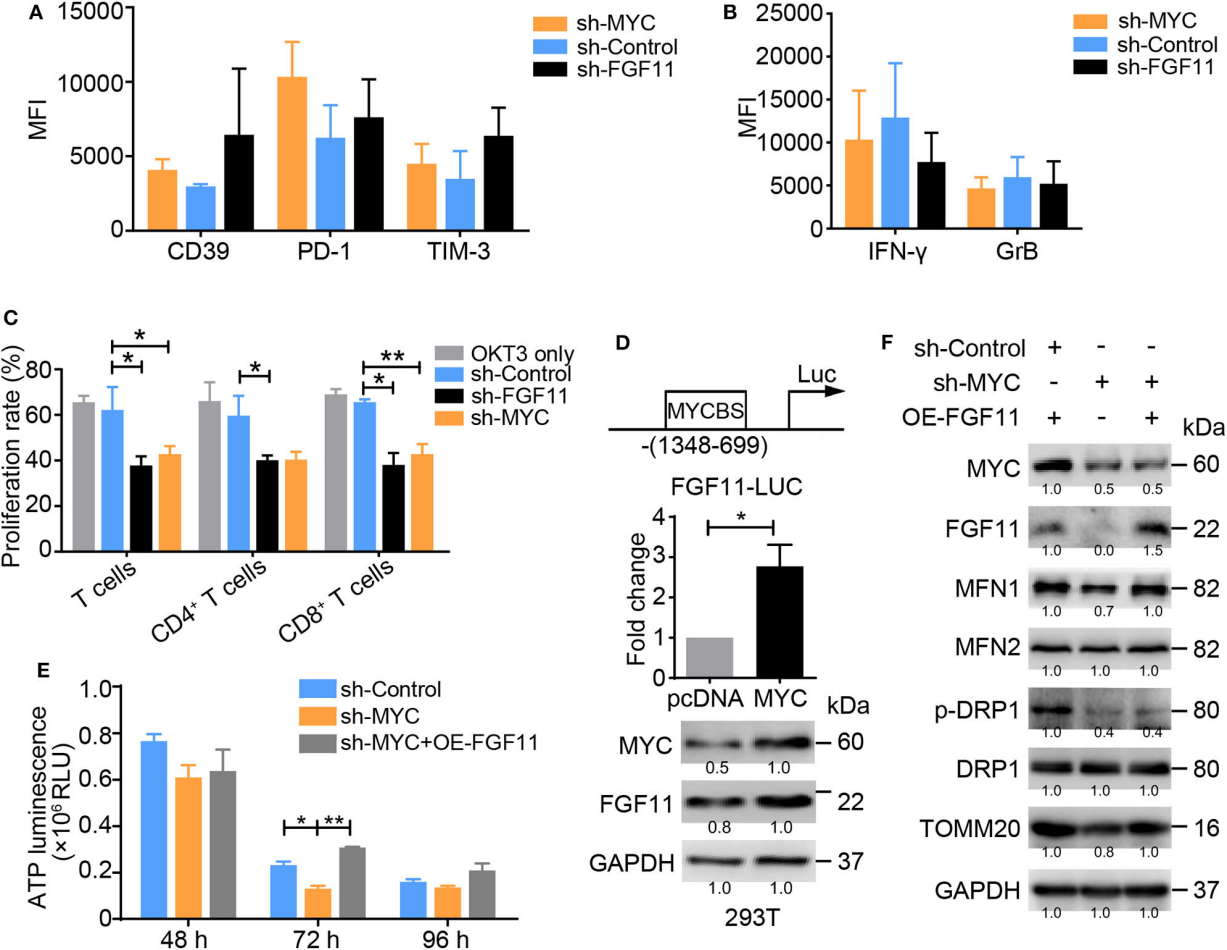
MYC transcriptionally activates FGF11 and MFN1 to trigger OXPHOS activity. **(A,B)** Graphs showing the expression levels of inhibitory molecules, including CD39, PD-1 and TIM-3, and the effector cytokines IFN-γ and GrB in activated T cells transduced with the shMYC, shFGF11, or shControl vector determined using flow cytometry. **(C)** Proliferation of activated T cells transfected with the shMYC, shFGF11, or shControl vector for 3 days. **(D)** Schematic showing the MYC-binding site (MYCBS) in the FGF11 promoter; a luciferase reporter assay was performed to assess the transcriptional regulation of FGF11 in 293T cells. **(E)** ATP production in activated T cells stimulated with the shControl, shMYC, or shMYC + FGF11 overexpression (OE) vector for 48, 72, and 96 h. **(F)** Immunoblot analysis of activated T cells cultured under the treatment conditions shown in **(E)** using the indicated antibodies; the data from one of three independent experiments are shown. All data were obtained from at least three independent experiments. The error bars represent the SEMs. *P < 0.05, **P < 0.01 (one-way ANOVA).

Intriguingly, the ablation of MYC or FGF11 in activated T cells resulted in increased levels of CD39, PD-1, and TIM-3, but decreased levels of IFN-γ and GrB and poor proliferation, consistent with their roles in the energy metabolism of activated T cells (Figures 6A–C). However, as shown in Figure 5, the effect of FGF11 on mitochondria function in activated T cells was less than MYC. Thus, we wondered whether FGF11 might serve as an additional signal transfer station between MYC and MFN1, supplementing the direct crosstalk between these molecules. We found that MYC bound to the *FGF11* promoter at a region including nucleotides −1348 to −699 and induced *FGF11* expression (Figure 6D). We subsequently introduced a lenti-FGF11 vector into shMYC-treated T cells and observed a reversal of the decrease in ATP production induced by MYC knockdown (Figure 6E). FGF11 overexpression reversed the decreases in MFN1 and TOMM20 levels, but not p-DRP1 levels, in shMYC-treated T cells (Figure 6F). These results support our hypothesis that FGF11 can act as a signal transducer between MYC and MFN1.

### The NPC Microenvironment Promotes an Increased Frequency of T Cells With the T_Exh_ Phenotype and Mitochondrial Fragmentation

**Figure 7:**
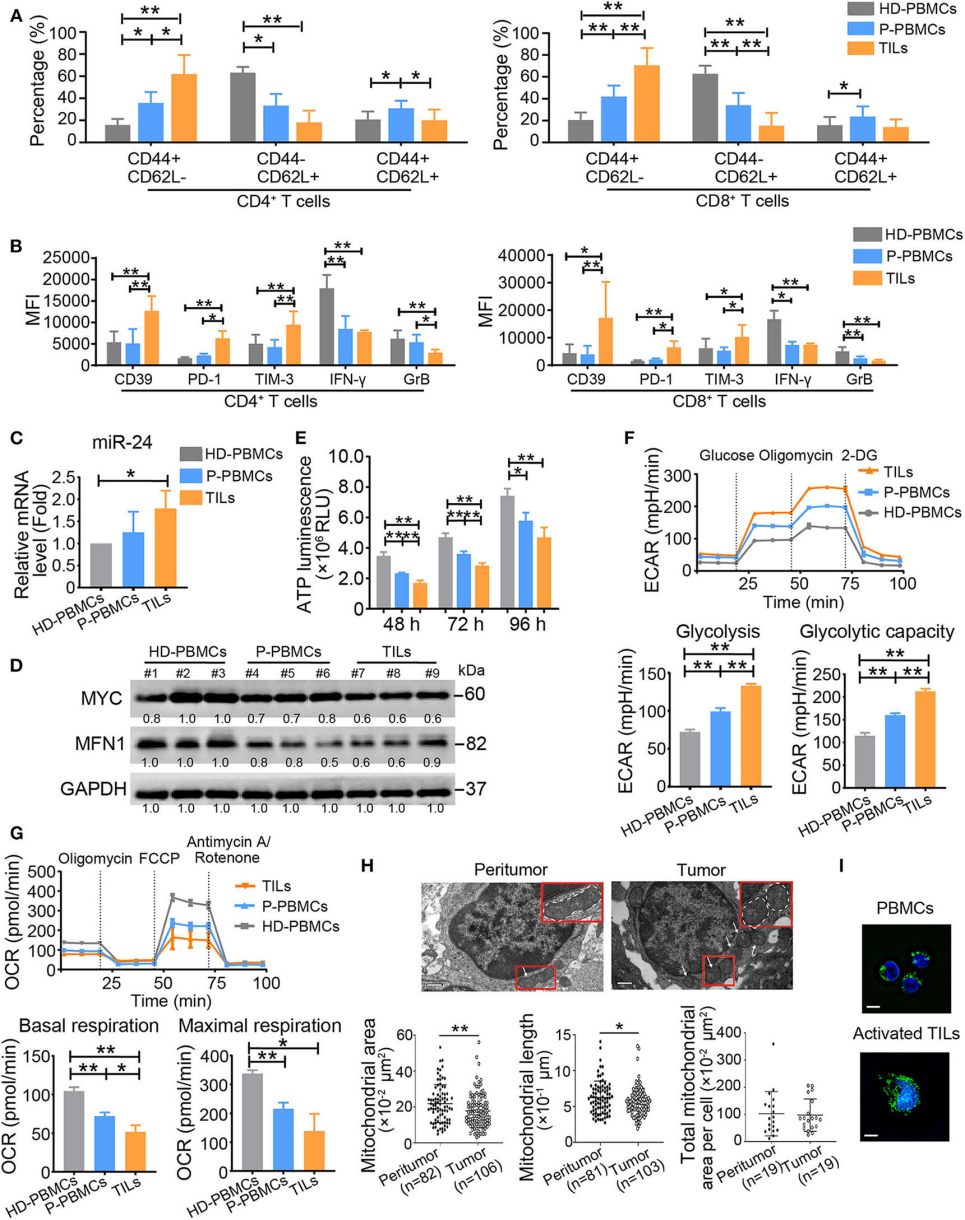
T_Exh_ frequency is increased in NPC patients. **(A)** PBMCs (P-PBMCs) and TILs were isolated from patients with NPC (*n* = 10). The percentages of different T cell subsets were calculated by performing staining for T cell surface markers, including CD4, CD8, CD44, and CD62L, followed by a FACS analysis. CD4^+^ (CD8^+^) CD44^+^CD62L^−^ cells were defined as T_eff_ cells, CD4^+^ (CD8^+^) CD44^−^CD62L^+^ cells were defined as naïve T cells, and CD4^+^ (CD8^+^) CD44^+^CD62L^+^ cells were defined as central memory T cells. PBMCs (HD-PBMCs) from age-matched HDs were included as controls (*n* = 10). **(B)** Summary of CD39, PD-1 and TIM-3 MFI levels in CD4^+^ and CD8^+^ P-PBMCs and TILs and HD-PBMCs (*n* = 10). **(C)** Real-time RT-qPCR assay showing the relative miR-24 levels in P-PBMCs, TILs and HD-PBMCs (*n* = 3) **(D)** Immunoblot analysis of P-PBMCs, TILs and HD-PBMCs using the indicated antibodies (*n* = 3). **(E)** ATP production in P-PBMCs, TILs and HD-PBMCs (*n* = 3). **(F,G)** The ECAR and OCR values in P-PBMCs, TILs, and HD-PBMCs (*n* = 3); the values were normalized to the number of cells. **(H)** Images of tumor and peritumor tissues from two patients. The images were acquired with a Gatan 1K × 1K charge-coupled device (CCD) camera (Gatan). Scale bar, 0.5 μm. **(I)** Image of mitochondrial staining in PBMCs and preactivated and activated TILs. The mitochondrial morphology of PBMCs and activated TILs was captured using a structured illumination microscope (Nikon). The mitochondria are green (MitoTracker Green), and the nuclei are blue (Hoechst). Scale bar, 50 μm. PBMCs = peripheral blood monocytes; TILs = tumor-infiltrating lymphocytes; MFI = mean fluorescence intensity. All the values are shown as the means ± SEMs. *P < 0.05, **P < 0.01 (one-way ANOVA or Mann–Whitney *U*-tests).

To determine whether our above-described results obtained with *in vitro*-activated T cells also occur *in vivo*, we analyzed circulating and tumor-infiltrating T cells from NPC patients and age-matched HDs by flow cytometry. This analysis revealed the same trends for the CD4^+^ and CD8^+^ T cell populations: TILs exhibited the highest percentage of the CD44^+^CD62L^−^ T_eff_ subset and the lowest proportion of CD44^−^CD62L^+^ naïve T cells and CD44^+^CD62L^+^ central memory T cells among the three groups of both CD4^+^ and CD8^+^ T cells (*P* < 0.01) (Figure 7A). However, compared with cells from HDs, T cells (including both CD4^+^ and CD8^+^ cells and particularly TILs) from patients with NPC expressed the inhibitory ligands CD39, PD-1, and TIM-3 at higher levels and the effector cytokines GrB and IFN-γ at lower levels (Figure 7B). These observations indicate that the T cells of NPC patients exhibit a T_Exh_ phenotype, particularly in tumor tissues, with lower proportions of naïve and central memory T cells than T cells of HDs. In addition, we observed increased expression of miR-24 in TILs compared with the PBMCs from patients with NPC and HDs, and decreased levels of MYC and MFN1 in the PBMCs and TILs from patients with NPC compared with HDs (Figures 7C,D). ATP production was decreased in patients with NPC, particularly in TILs (Figure 7E). Both PBMCs and TILs from patients with NPC displayed a significantly higher ECAR value and decreased OCR value (Figures 7F,G). We subsequently assessed the mitochondrial morphology of TILs and infiltrated lymphocytes in peritumor tissues by transmission electron microscopy (TEM). The average area and length of mitochondria in TILs were less than the mitochondria in lymphocytes that infiltrated peritumor tissues, although the total mitochondria numbers per cell were similar in these samples (Figure 7H). However, mitochondrial tubulation was induced by the reactivation of TILs *in vitro* (Figure 7I). These data suggest that the T_Exh_ phenotype is associated with the dysregulation of mitochondrial dynamics in NPC microenvironments.

### Inhibiting the Development of the T_Exh_ Phenotype by Inhibiting MiR-24 Signaling Decreases NPC Tumorigenesis in Nude Mice

**Figure 8:**
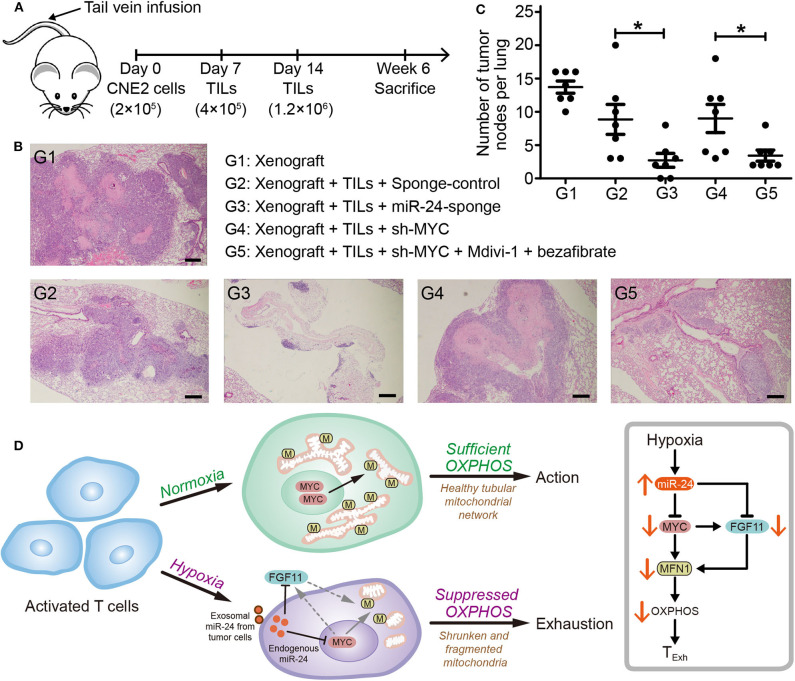
Inhibition of the T_Exh_ status of TILs reduces the growth of NPC xenografts in mice. **(A)** Nude mice were injected with 2 × 10^5^ CNE2 cells via the tail vein, and 1 and 2 weeks later, the mice received injections of TILs into the tail vein (4 × 10^5^ and 1.2 × 10^6^ cells, respectively). The TILs were pretreated *in vitro* for 3 days as follows: G1 (*n* = 5), xenograft + PBS; G2 (*n* = 5), xenograft + TILs administered the lenti-sponge-control vector; G3 (*n* = 5), xenograft + TILs administered the lenti-miR-24-sponge vector; G4 (*n* = 6), xenograft + TILs administered the lenti-shMYC vector; and G5 (*n* = 5), xenograft + TILs administered the lenti-shMYC vector, Mdivi-1, and bezafibrate. **(B)** Images of H&E staining of the lungs of mice in the different treatment groups (G1 to G5). Scale bar, 250 μm. **(C)** Statistical analysis of the numbers of tumor nodes in the lungs of mice in the various groups (G1 to G5); the nodes were counted in each 10x microscopic field. The data are presented as the means ± SEMs. *P < 0.05 (one-way ANOVA). **(D)** Cartoon schematic showing the regulation of T_Exh_ under hypoxic conditions via the miR-24 signaling-mediated reprogramming of mitochondrial energy metabolism.

To determine whether inhibition of miR-24-induced T_Exh_ restricts the progression of NPC *in vivo*, we implemented an experimental pipeline based on NPC xenografts in nude mice as described previously (26) (Figure 8A). Both the tumor node number and size were decreased by pretreatment of TILs with miR-24 sponges prior to their injection into xenograft mice. The activity of TILs was rescued by the coadministration of Mdivi-1, an efficacious inhibitor of the mitochondrial fission protein DRP1, and bezafibrate, an approved lipid-lowering drug that is presumed to prevent mitochondrial dysfunction (Figures 8B,C). Based on these results, the expression levels of miR-24 in TILs are closely associated with the tumor-inhibiting activity, and boosting mitochondrial biogenesis and fusion promotes the response to MYC-depleted TILs in mice. This association might be established based on (and effectively influenced by) the health of mitochondria and the resulting OXPHOS capacity.

## Discussion

In this study, we found that hypoxia stress inhibits mitochondrial fusion in activated T cells and subsequently induces the T_Exh_ phenotype by disrupting mitochondrial function. Hypoxia upregulates miR-24 in tumor cells and TILs. Endogenous and exogenous miR-24 suppresses the expression of MYC and FGF11 in TILs and thereby decreases the cellular OXPHOS level by disrupting MFN1-mediated mitochondrial fusion (Figure 8D). Importantly, we found that inhibition of miR-24/MYC expression and/or promotion of mitochondrial fusion can restore the T_Exh_ status of TILs from NPC patients and restrict NPC tumorigenesis in xenograft mice. These results highlight a novel pathway for tumor microenvironment-induced T_Exh_ and provide new potential targets for cancer immunotherapy.

The hypoxic conditions in the TME lead to various effects that are thought to promote tumor progression. Specifically, hypoxia triggers the upregulation of PD-L1 in tumor cells in a HIF-1α-dependent manner (27), and HIF-1α accumulation in T cells has been suggested to favor differentiation into T_H_17 cells and to promote inflammatory diseases in humans (28, 29). Although these HIF-1α-mediated processes might contribute to T cell differentiation and dysfunction in the TME, how activated TILs directly respond to hypoxia and the role played by hypoxia in the development of the T_Exh_ phenotype remain unclear. We observed increased HIF-1α levels in activated T cells derived from PBMCs under hypoxic conditions. However, enhancement of HIF-1α activity might promote the glycolytic metabolism and effector function of CD8^+^ T cells in response to persistent antigen exposure (30). Hypoxia reportedly induces the expression of effector molecules, including GrB, via a HIF-dependent mechanism (31, 32). These results appear somewhat inconsistent with our data, which showed that hypoxia inhibits the effector function of activated T cells. Furthermore, hypoxia reduced the mitochondrial metabolism and mass in the present study, consistent with the observations of the status of mitochondrial metabolism in the tumor microenvironment containing dysfunctional T cells or exhausted T cells (33, 34). We reason that miR-24-induced MYC downregulation plays a more important role than other processes in the development of the T_Exh_ phenotype in the TME. In addition, a recent study found that HIF-1α induces miR-24 expression in colorectal cancer cells under hypoxic stress (35). Thus, miR-24 upregulation in T cells might also be related to HIF-1α stabilization.

Notably, miR-24 belongs to the miR-23/27/24 family, which contains multiple members and two paralogs in humans: miR-23a/27a/24-2 (the miR-23a cluster) on chromosome 19 and miR-23b/27b/24-1 (the miR-23b cluster) on chromosome 9 (36). According to previous studies, miR-24 is actively involved in T cell regulation. For instance, the miR-23~27~24 cluster is upregulated during postthymic CD8^+^ T cell differentiation and sensitizes human CD8^+^CD28^−^ effector memory T cells to apoptotic death by suppressing histone variant H2AX expression (37, 38). In tumor microenvironments, miRs, including miR-24, play important roles in tumor immune editing by regulating target gene expression in a number of cell signaling pathways (39, 40). Here, we showed that under hypoxic conditions, TILs might accumulate miR-24 through both directly induced endogenous expression and import from T-EXOs. Thus, high loads of miR-24 can effectively induce metabolic and proliferative alterations in activated T cells. Although miR-24 induced mitochondrial dysfunction, as indicated by decreases in ATP production and OXPHOS activity (Figure 3), the RNA-seq transcriptome data indicated that most OXPHOS-related genes were upregulated following hypoxia or miR-24 overexpression in response to mitochondrial damage (Figure 4). These seemingly controversial results might be explained by a protective feedback mechanism of cells under stress. Similar regulatory mechanisms for mitochondrial metabolic reprogramming have been observed in T cells under hypoxia (41, 42).

Our study revealed that MYC is a major target of miR-24 in T cells. MYC is required at the early stage of T cell activation that precedes cell cycle entry (43). It has also been suggested that MYC promotes effector functions in CD8^+^ T cells (44) and is essential for the expression of genes involved in metabolic reprogramming during T cell activation (45). Given its roles in activated T cells, ablation of MYC might be closely related to T_Exh_ in the TME. We supported this possibility by showing that MYC directly impacts mitochondrial dynamics through control of the expression of the mitochondrial fusogen MFN1. Similarly, a study on mammary epithelial cells found that MYC overexpression promotes mitochondrial fusion via the mitochondrial outer membrane-anchored phospholipase PLD6 (46). As shown in our experiments, mitochondrial fusion is intimately coupled with OXPHOS activity and ATP production in activated T cells. Recent studies have also proposed that mitochondrial dynamics are able to control T cell fate through metabolic programming because enforcing mitochondrial fusion in activated T_eff_ cells imposes characteristics of memory T cells and enhances antitumor function (47). A change in mitochondrial morphology has been proposed as a mechanism for bioenergetic adaptation to metabolic demands (48). In addition, we observed that the expression level of PGC1α remained unchanged and relatively low in activated T cells with and without miR-24 overexpression, which indicates that the miR-24-induced changes in the mitochondrial architecture are not dependent on PGC1α-mediated mitochondrial biogenesis. In contrast, although MFN1 has been found to share some functional redundancy with MFN2 in mouse epithelial fibroblast (MEF)-based assays (20), ablation of MFN1 in HeLa cells is sufficient to induce mitochondrial fragmentation (49). Thus, the disruption of mitochondrial fusion caused by miR-24-induced downregulation of the MYC-MFN1 pathway is likely an event upstream of metabolic reprogramming and a major driving force promoting T_Exh_ in the TME, which is considered a hypoxic and extremely nutrient-deprived environment. Notably, FGF11 plays an interesting role in this process. On the one hand, FGF11 responds to miR-24 upregulation both directly and through MYC; on the other hand, FGF11 and MYC both regulate the expression of MFN1. Thus, miR-24 controls MFN1-mediated mitochondrial dynamics in a “dual switch mode.”

T_Exh_ in the TME is a major barrier to the success of immunotherapies for solid cancers, such as CAR-T cell and PD-1 antibody therapies. Here, we found that increases in the frequency of the T_Exh_ phenotype among circulating T cells and TILs from NPC patients occurred concomitantly with decreases in mitochondrial mass and fusion deficiency in the TME. Although we have previously investigated the safety of and the objective clinical response to autologous *in vivo*-activated TILs in patients with late-stage NPC in a phase I study (50), we noted that the mitochondria in these TILs displayed a healthy tubular network (Figure 7I), which would enable efficient OXPHOS. A previous study based on mouse-derived T_eff_ cells found that enforcement of mitochondrial fusion improves adoptive cellular immunotherapy against tumors (47). In this study, we found that targeting of miR-24 signaling or promotion of mitochondrial biogenesis and fusion enhanced the antitumor activity of human TILs in xenograft mice, which demonstrates the effectiveness of regulating mitochondrial dynamics in promoting the effector functions of human T cells. Therefore, targeting the miR-24 or MYC/FGF11-MFN1 pathway could be a potential effective approach for correcting mitochondrial defects in exhausted T cells and restoring healthy metabolism. This approach might prevent the development of the T_Exh_ phenotype or reverse the exhausted fate of TILs and could eventually improve the clinical efficacy of adaptive T cell therapies against solid tumors.

## Data Availability Statement

The datasets presented in this study can be found in online repositories. The names of the repository/repositories and accession number(s) can be found in the article/Supplementary Material.

## Ethics Statement

The fresh tumor specimens and peripheral blood samples were collected at Sun Yat-sen University Cancer Center, Guangzhou, China, in 2015. The study was approved by the Research Ethics Committee of Sun Yat-sen University Cancer Center (GZR2013-040). The patients/participants provided their written informed consent to participate in this study. All animal experiments were performed in accordance with protocols approved by the Institutional Animal Care and Use Committee of Sun Yat-sen University, Guangzhou, China (L102012018060P). Written informed consent was obtained from the individual(s) for the publication of any potentially identifiable images or data included in this article.

## Author Contributions

JL and SG proposed the concept and conceived the entire study. Y-NL, D-JH, J-FY, H-HN, C-XZ, JH, J-MG, and H-XC performed the experiments. H-QM, Q-YC, JH, and X-SZ selected the patients and collected the clinical samples. JL, SG, and Y-NL wrote the manuscript. JL supervised the entire project. All authors contributed to the article and approved the submitted version.

## Conflict of Interest

The authors declare that the research was conducted in the absence of any commercial or financial relationships that could be construed as a potential conflict of interest.
