# Supplementary material for: Hypoxia Induces Mitochondrial Defect That Promotes T Cell Exhaustion in Tumor Microenvironment Through MYC-Regulated Pathways
Source: Front Immunol. 2020 Aug 21;11:1906. doi: 10.3389/fimmu.2020.01906 (PMC7472844; doi:10.3389/fimmu.2020.01906)
Supplement: Supplementary file 1 [file Data_Sheet_1.docx]

**SUPPLEMENTAL MATERIALS**

**Summary:** The Supplemental Materials file includes 2 supplementary figures and figure legends, 3 supplementary tables (clinical information for patients with NPC, reagents and resources, and list of abbreviations), supplemental experimental procedures and references.

**SUPPLEMENTARY FIGURES**

**Supplementary Figure 1**

**
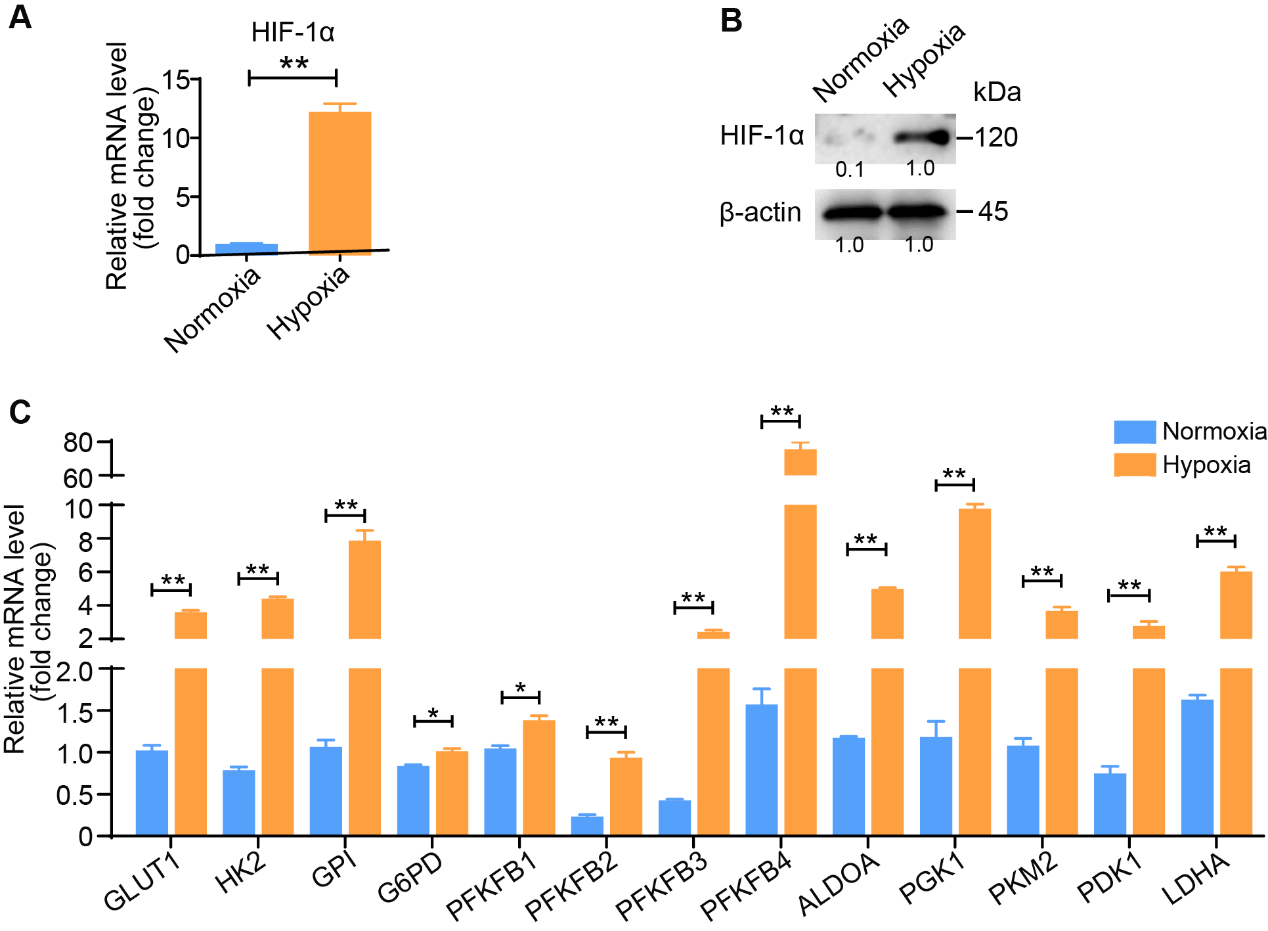
**

**Figure 1** Hypoxia inhibits glycolysis and mitochondrial OXPHOS metabolism in activated T cells *in vitro*. The data presented in this figure are related to the results presented in Figure 1. **(A-B)** Levels of the HIF-1α mRNA and protein in activated T cells cultured under normoxic and hypoxic conditions. **(C)** The relative mRNA levels of glycolytic genes in OKT3-stimulated T cells under normoxic and hypoxic conditions were measured using qRT-PCR. All values are presented as means ± SEM; * P < 0.05, ** P < 0.01.

**Supplementary Figure 2**

**
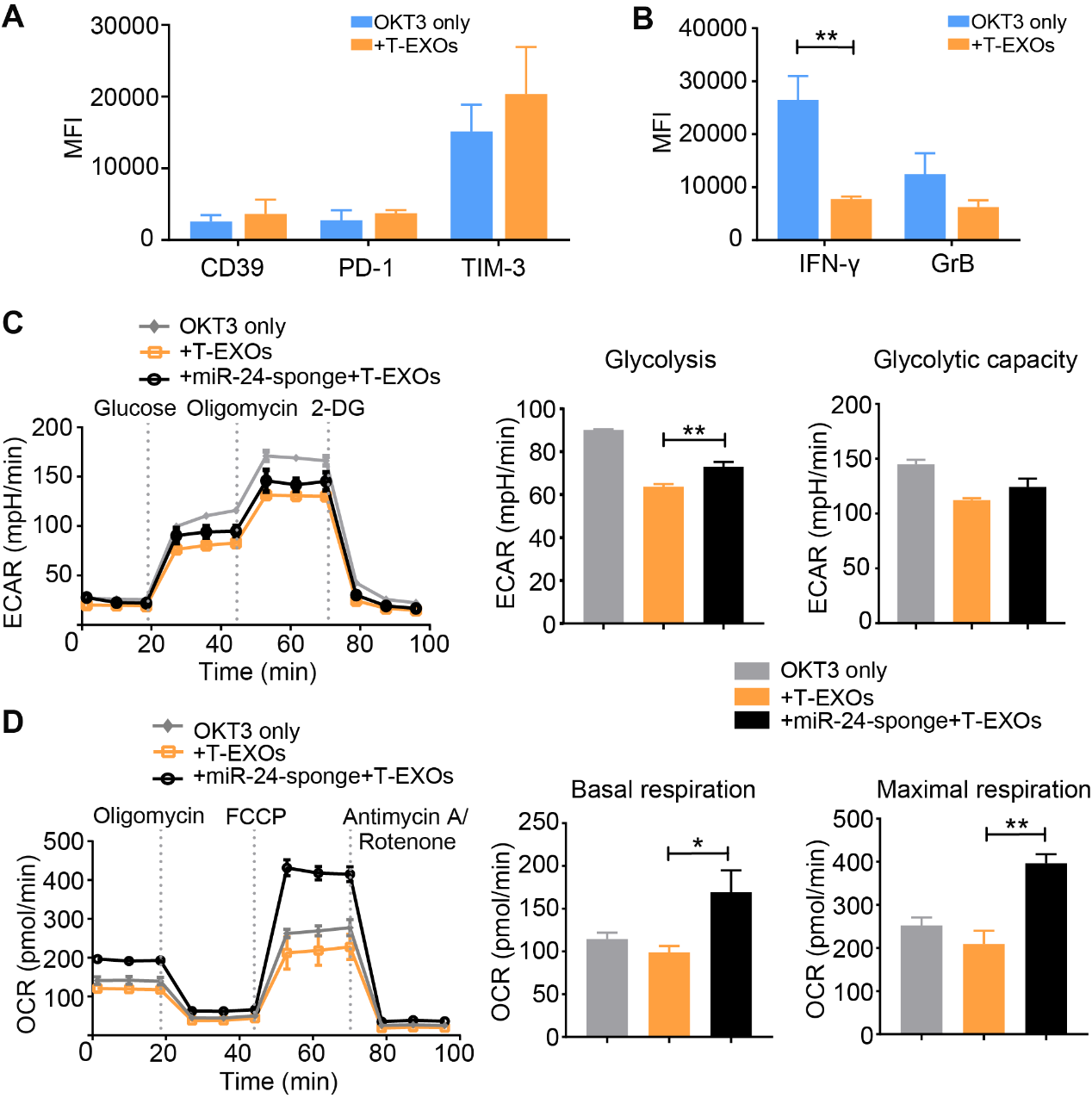
**

**Figure 2** Exosomal miR-24 induces the T_Exh_ phenotype and mitochondrial energy metabolism reprogramming in activated T cells. These data are related to the results shown in Figure 3. **(A-B)** Representative histograms of the CD39, PD-1, TIM-3, IFN-γ and GrB levels detected in OKT3-stimulated T cells cultured in the presence or absence of T-EXOs. **(C-D)** The ECAR and OCR values of OKT3-stimulated T cells cultured in the absence or presence of miR-24/T-EXOs and miR-24-sponge/T-EXOs were measured.

**SUPPLEMENTAL TABLES**

**Table S1.** Clinical information of patients with NPC.

| **No.** | **Sex** | **Age (y)** | **Stage** |
| --- | --- | --- | --- |
| NPC-P1 | F | 33 | T3N2M0 |
| NPC-P2 | F | 42 | T4N1M0 |
| NPC-P3 | M | 37 | T3N3M0 |
| NPC-P4 | M | 49 | T3N2M0 |
| NPC-P5 | M | 41 | T3N1M0 |
| NPC-P6 | M | 56 | T3N1M0 |
| NPC-P7 | M | 37 | T4N2M0 |
| NPC-P8 | F | 41 | T3N1M0 |
| NPC-P9 | F | 53 | T3N3M0 |
| NPC-P10 | F | 59 | T3N3M0 |

**Table S2.** Reagents and resources.

| **Antibody** | **Source** | **Identifier** | **Location** |
| --- | --- | --- | --- |
| Anti-human CD39 FITC | eBioscience | REF#11-0399-42 | San Diego, CA, USA |
| Anti-human CD39 PE-Cy7 | eBioscience | REF#25-0399-41 | San Diego, CA, USA |
| PE-mouse anti-human CD279 (PD-1) | BD Biosciences | CAT#560795 | San Jose, CA, USA |
| Alexa Fluor 647 mouse anti-human TIM3 | BD Biosciences | CAT#565558 | San Jose, CA, USA |
| Anti-human IFN-γ APC | eBioscience | REF#17-7319-82 | San Diego, CA, USA |
| FITC mouse anti-human GrB | eBioscience | CAT#560211 | San Jose, CA, USA |
| Anti-human GrB PE | BD Biosciences | REF#12-8899-41 | San Diego, CA, USA |
| Anti-human/mouse CD44 FITC | eBioscience | REF#11-0441-82 | San Diego, CA, USA |
| Anti-human/mouse CD44 PE-Cyanine 7 | eBioscience | REF#25-0441-82 | San Diego, CA, USA |
| Anti-human CD62L APC | eBioscience | REF#17-0629-42 | San Diego, CA, USA |
| Anti-human CD4 PE-Cyanine 7 | eBioscience | REF#25-0049-42 | San Diego, CA, USA |
| Anti-human CD8a PE | eBioscience | REF#12-0086-42 | San Diego, CA, USA |
| Anti-human HLA-A2 APC | eBioscience | REF#17-9876-42 | San Diego, CA, USA |
| 7-AAD | BD Biosciences | CAT#51-68981E | San Jose, CA, USA |
| pAb anti-GLUT1 immunogen affinity-purified rabbit | Novus Biologicals | CAT#NB110-39113 | Littleton, CO, USA |
| Rb mAb to TOMM20 | Abcam | LOT#GR163072-5 | Cambridge, UK |
| c-MYC mouse monoclonal IgG | Santa Cruz Biotechnology | LOT#I1415 | Paso Robles, CA, USA |
| Rb pAb to FGF11 | Abcam | LOT#GR94111-1 | Cambridge, UK |
| Mouse anti-human LMP1 | Gene Tech | CAT#GM089729 | Shanghai, China |
| GAPDH rabbit polyAb | Proteintech | CAT#10494-1-Ap | Wu Han, HB, China |
| Peroxidase-conjugated AffiniPure goat anti-mouse IgG | Proteintech | CAT#SA00001-1 | Wu Han, HB, China |

| **Reagent** | **Source** | | **Identifier** | | **Location** | |
| --- | --- | --- | --- | --- | --- | --- |
| RPMI medium 1640 basic (1X) | | Gibco | | LOT#8117203 | | Suzhou, JS, China |
| X-VIVO-15 medium | | Lonza | | CAT#04-418Q | | Walkersville, MD, USA |
| DMEM basic | | Gibco | | LOT#2117207 | | Suzhou, JS, China |
| Collagenase type IV | | Sigma-Aldrich | | CAT#C5138-MG100 | | St. Louis, MO, USA |
| Interleukin-2 | | Beijing Four Rings | | CAT#S10970017 | | Beijing, China |
| Anti-hCD3-purified mouse monoclonal IgG1 | | R&D Systems | | CAT#MAB100 | | Minneapolis, MN, USA |
| Polybrene | | Abbott Laboratories Corp | | LOT#SC-134220 | | Chicago, Illinois, USA |
| TRIzol reagent | | Invitrogen | | REF#15596026 | | Carlsbad, CA, USA |
| RevertAid First-Strand cDNA Synthesis Kit | | Thermo Scientific | | CAT#K1622 | | Waltham, MA, USA |
| ChamQ SYBR qPCR Master Mix | | Vazyme Biotechnology | | LOT#Q311-02 | | Nanjing, JS, China |
| CFSE proliferation dye | | eBioscience | | REF#65-0850-85 | | San Diego, CA, USA |
| 2-DG | | Sigma-Aldrich | | LOT#WXBC1593V | | St. Louis, MO, USA |
| Oligomycin | | Seahorse Bioscience | | CAT#103015-100 | | North Billerica, MA, USA |
| Carbonyl cyanide-4 phenylhydrazone (FCCP) | | Seahorse Bioscience | | CAT#103015-100 | | North Billerica, MA, USA |
| Rotenone/antimycin A | | Seahorse Bioscience | | CAT#103015-100 | | North Billerica, MA, USA |
| XF Base medium minimal DMEM | | Seahorse Bioscience | | LOT#30815011 | | North Billerica, MA, USA |
| XF calibrant | | Seahorse Bioscience | | LOT#085 | | North Billerica, MA, USA |
| MitoTracker Green | | Thermo Scientific | | CAT#M7514 | | Waltham, MA, USA |
| MitoTracker Deep Red | | Thermo Scientific | | CAT#M22426 | | Waltham, MA, USA |
| Poly-D-lysine | | Sigma-Aldrich | | CAT#P6407 | | St. Louis, MO, USA |
| CellTiter-Glo Luminescent Cell Viability Assay | | Promega | | REF#G7570 | | Madison, WI, USA |

| **Software and Algorithm** | | **Source** | | | **Identifier** | **Location** | |
| --- | --- | --- | --- | --- | --- | --- | --- |
| FlowJo V10 | | | FlowJo, LLC | | https://www.flowjo.com/ | | Ashland, Oregon |
| KOBAS 2.0 | | | Bioinformatics Center, Peking University | | http://kobas.cbi.pku.edu.cn/ | | Beijing, China |
| NIS-Elements Viewer 4.50 | | | Nikon Instruments software | | https://www.nikoninstruments.com | | Tokyo, Japan |
| GraphPad Prism 6 | | | GraphPad software | | https://www.graphpad.com/scientific-software/prism/ | | La Jolla, CA, USA |
| Image Lab | | | Bio-Rad Laboratories | | http://www.bio-rad.com/en-us/product/image-lab-software | | Hercules, CA, USA |
| IBM SPSS Statistics 19.0 | | | IBM Analysis | | https://www.ibm.com/analytics/data-science/predictive-analytics/spss-statistical-software | | Chicago, IL, USA |
| Adobe Photoshop CS6 | | | Adobe Systems | | https://www.adobe.com | | San Jose, CA, USA |
| **Primers Used for qRT-PCR** | | | | | | | |
| **Gene** | **Forward** | | | **Reverse** | | | |
| *MYC* | 5’-GGCTCCTGGCAAAAGGTCA-3ˈ | | | 5ˈ-CTGCGTAGTTGTGCTGATGT-3ˈ | | | |
| *FGF11* | 5ˈ-CTGTACGCCTCTGCTCTCTAC-3ˈ | | | 5ˈ-GCCTTGGTCTTCTTAACTCGGT-3ˈ | | | |
| *GLUT1* | 5ˈ-ATTGGCTCCGGTATCGTCAAC-3ˈ | | | 5ˈ-GCTCAGATAGGACATCCAGGGTA-3ˈ | | | |
| *HK2* | 5ˈ-TGCCACCAGACTAAACTAGACG-3ˈ | | | 5ˈ-CCCGTGCCCACAATGAGAC-3ˈ | | | |
| *GPI* | 5ˈ-CCGCGTCTGGTATGTCTCC-3ˈ | | | 5ˈ-CCTGGGTAGTAAAGGTCTTGGA-3ˈ | | | |
| *G6PD* | 5ˈ-CGAGGCCGTCACCAAGAAC-3ˈ | | | 5ˈ-GTAGTGGTCGATGCGGTAGA-3ˈ | | | |
| *PFKFB1* | 5ˈ-GGTGCCCGTGTCTTCTTTGT-3ˈ | | | 5ˈ-AAGCATCATCGAAACGCTCTC-3ˈ | | | |
| *PFKFB2* | 5ˈ-AGTCCTACGACTTCTTTCGGC-3ˈ | | | 5ˈ-TCTCCTCAGTGAGATACGCCT-3ˈ | | | |
| *PFKFB3* | 5ˈ-ATTGCGGTTTTCGATGCCAC-3ˈ | | | 5ˈ-GCCACAACTGTAGGGTCGT-3ˈ | | | |
| *PFKFB4* | 5ˈ-TCCCCACGGGAATTGACAC-3ˈ | | | 5ˈ-GGGCACACCAATCCAGTTCA-3ˈ | | | |
| *ALDOA* | 5ˈ-ATGCCCTACCAATATCCAGCA-3ˈ | | | 5ˈ-GCTCCCAGTGGACTCATCTG-3ˈ | | | |
| *PGK1* | 5ˈ-GACCTAATGTCCAAAGCTGAGAA-3ˈ | | | 5ˈ-CAGCAGGTATGCCAGAAGCC-3ˈ | | | |
| *PKM2* | 5ˈ-ATGTCGAAGCCCCATAGTGAA-3ˈ | | | 5ˈ-TGGGTGGTGAATCAATGTCCA-3ˈ | | | |
| *PDK1* | 5ˈ-GAGAGCCACTATGGAACACCA-3ˈ | | | 5ˈ-GGAGGTCTCAACACGAGGT-3ˈ | | | |
| *LDHA* | 5ˈ-TCTCTGTAGCAGATTTGGCAGA-3ˈ | | | 5ˈ-AAGACATCATCCTTTATTCCGTAA-3ˈ | | | |
| *GAPDH* | 5ˈ-CGAGATCCCTCCAAAATCAAGTGGGG-3ˈ | | | 5ˈ-ACACGTTGGCAGTGGGGACAC-3ˈ | | | |

| **Primers Used for Plasmid Construction** | | |
| --- | --- | --- |
|  | | |
| **Plasmid** | **Forward** | **Reverse** |
| MYC-pcDNA3.1 | 5’-TTTAAACTTAAGCTTGGTACCGCCACCCTGGATTTTTTTCGGGTAGTGGAA-3’ | 5’-AACGGGCCCTCTAGACTCGAGTCACTTATCGTCGTCATCCTTGTAATCGAATTC-3’ |
| FGF11-report luc | 5’-CCCCATTCCAGCCTCGCTATT-3’ | 5’-GGCAGCGGCAGCGGCTGTGGGAAG-3’ |
| pSin-EF2-puro-FGF11 | 5’-CCCGGACGAATTCTTCGAAATGTCTCTCTCTCCAGAGCCT-3’ | 5’-TGCGGATCACTAGTGCTAGCTCAGGGGGCAGGGGGACT-3’ |

**Table S3.** List of abbreviations.

| **Abbreviations** | **Full names** |
| --- | --- |
| NPC | Nasopharyngeal Carcinoma |
| T_Exh_ | T Cell Exhaustion |
| PBMC | Peripheral Blood Mononuclear Cell |
| TIL | Tumor-infiltrating Lymphocyte |
| T-EXO | Exosome from Tumor Cell Lines |
| FGF11 | Fibroblast Growth Factor 11 |
| OXPHOS | Oxidative Phosphorylation |
| PD-1 | Programmed Cell Death Protein 1 |
| TIM-3 | T Cell Immunoglobulin Mucin-3 |
| GrB | Granzyme B |
| IFN-γ | Interferon-γ |
| PGC1ɑ | Proliferator-activated Receptor Gamma Coactivator 1-alpha |
| MFN1 | Mitofusin 1 |
| MFN2 | Mitofusin 2 |
| DRP1 | Dynamin—related Protein1 |
| TOMM20 | Translocase of Outer Mitochondrial Membrane 20 Homolog |
| 293T | HEK293T Human Embryonic Kidney Cells |
| CFSE | 5,6-Carboxyfluorescein Diacetate Succinimidyl Ester |
| ECAR | Extracellular Acidification Rate |
| OCR | Oxygen Consumption Rate |
| 2-DG | 2-Deoxy-D-glucose |
| FCCP | Carbonyl Cyanide-4 Phenylhydrazone |
| GLUT1 | Glucose Transporter 1 |
| HK2 | Hexokinase 2 |
| GPI | Phosphohexose Isomerase |
| G6PD | Glucose-6-phosphate Dehydrogenase |
| PFKFB1 | 6-Phosphofructokinase-1 |
| PFKFB2 | 6-Phosphofructo-2-kinase |
| PFKFB3 | 6-Phosphofructo-3-kinase |
| PFKFB4 | 6-Phosphofructo-4-kinase |
| ALDOA | Aldolase |
| PGK1 | Phosphoglycerate Kinase |
| PKM2 | Pyruvate Kinase M2 |
| PDK1 | 3-Phosphoinositide-dependent Protein Kinase-1 |
| LDHA | Lactate Dehydrogenase A |
| GAPDH | Glyceraldehyde 3-Phosphate Dehydrogenase |

**SUPPLEMENTAL EXPERIMENTAL PROCEDURES**

**Sample Collection and T Cell Culture**

Some of the cells were cultured in 24-well plates for 3 days with X-VIVO-15 medium containing recombinant human IL-2 (10 IU/ml) to ensure that we would be able to collect a sufficient number of T cells for further experiments. Other cells were maintained in X-VIVO-15 medium containing IL-2 (150 IU/ml) for 3-4 weeks to obtain purified T cells, and a previously described rapid T cell expansion protocol was used (1). Peripheral blood mononuclear cells (PBMCs) were isolated from peripheral blood obtained from patients with NPC or HDs using Ficoll density gradient centrifugation, and then frozen until use in the *in vitro* proliferation and differentiation analyses. PBMCs were plated in 48- or 24-well plates coated with OKT3 (1 µg/ml) and cultured with low-dose rhIL-2 (150 IU/ml) medium for various treatments. For the experiments conducted under hypoxic conditions, the T cells were incubated in the presence of 1% O_2_, 5% CO_2,_ 9% H_2_ and 85% nitrogen at 37°C for 48 h.

The NPC cell lines TW03 and CNE2 were maintained in our laboratory. Exosomes were isolated from the culture supernatants of TW03 (T-EXO) and miR-24-sponge-treated TW03 cells (miR-24-sponge-T-EXO) by ultracentrifugation, as previously described (2). Then, the T-EXOs (10 µg/ml) and miR-24-sponge-T-EXOs (10 µg/ml) were used to treat the OKT3-stimulated PBMCs. After 48 h, the PBMCs treated with T-EXOs or miR-24-sponge-T-EXOs were used for ECAR and OCR measurements.

**Plasmid Construction, Lentivirus Production and Transduction**

To generate recombinant lentiviruses, a lentiviral expression construct and a packaging plasmid mix were cotransfected into 293T cells according to the manufacturer’s instructions. T cells were infected with recombinant lentiviruses for transduction and treated with 8 μg/ml polybrene (Abbott Laboratories Corp, Abbott Park, IL, USA). Vectors expressing either the c-MYC shRNA or scrambled shRNA were generated using a Sigma shRNA system according to the manufacturer’s instructions. The target sequence of the human c-MYC shRNA is 5’-CCTGAGACAGATCAGCAACAA-3’.

**T Cell Proliferation and Differentiation Assay**

The PBMCs from HDs were labeled with 10 μM carboxyfluorescein diacetate succinimidyl ester, plated in OKT3-coated 96-well plates, infected with recombinant lentiviruses for transduction, and cultured for 3 days. The cells were harvested and detected using flow cytometry, and data were acquired with a flow cytometer. PBMCs were cultured in an OKT3-coated plate for 2 to 3 days under normoxic or hypoxic conditions to mimic T cell receptor (TCR)-activated T cell differentiation in our *in vitro* experiments. The effector T cell (T_eff_) or naïve T cell population was gated based on surface markers, including CD44 and CD62L. Suppressive markers, including PD-1, CD39 and TIM-3, and the cytokines IFN-γ and granzyme B (GrB) were included in the analysis of the T_Exh_ cell phenotype. Briefly, for intracellular FACS staining, cells were stimulated with a leukocyte activation cocktail that contained phorbol 12-myristate-13-acetate (PMA), ionomycin, brefeldin A, and BD GolgiPlug in an incubator at 37°C with a 5% CO_2_ atmosphere for 4 h. After stimulation, the cells were washed and stained for surface phenotype markers for 20 min on ice. After permeabilization and fixation, the cells were intracellularly stained with anti-IFN-γ and anti-GrB antibodies. The cells were then detected using a Beckman Coulter Gallios flow cytometer and analyzed with FlowJo V10 software.

**RNA Sequencing (RNA-seq) and Analysis**

Total RNA was extracted from lentivirus-transduced T cells using TRIzol reagent and dissolved in RNase-free water. RNA-seq and analysis were conducted by RiboBio. A gene set enrichment analysis (GSEA) based on canonical pathways was performed as previously described (3). Briefly, the normalized expression data were analyzed and visualized with GSEA software (version 3.0, http://www.broadinstitute.org/gsea/). The raw read counts per locus were normalized across the samples, and the differential gene expression data were analyzed using DEseq.

**Real-Time RT-qPCR and Immunoblotting**

Total RNA was extracted from T cells using TRIzol reagent according to the manufacturer’s instructions. RT-PCR was performed using a RevertAid First-Strand cDNA Synthesis Kit and qPCR SYBR Master Mix, as described previously (2). The PCR primer sequences are listed in Supplemental Materials: Table S2. A miR-24 assay was performed using stem-loop primers purchased from RiboBio, and U6 small nuclear RNA (RiboBio) was used for normalization. For immunoblotting, cell lysates were separated using sodium dodecyl sulfate polyacrylamide gel electrophoresis (SDS-PAGE), and proteins were transferred to polyvinylidene fluoride (PVDF) membranes. The membranes were blocked and sequentially incubated with the indicated primary antibodies (as listed in **Supplemental Materials: Table S2**) and horseradish peroxidase (HRP)-conjugated secondary antibodies.

**Luciferase Reporter Assay**

The full-length cDNA encoding the *c-MYC* gene was obtained from human 293T cells using PCR and cloned into the pcDNA3.1 plasmid. The *FGF11* promoter region (nucleotides -1348 to -699) was cloned upstream of the Gaussia luciferase gene in the pEZX-MT05 vector, and the TK-luc plasmid (Promega) was included as a control. The MYC-pcDNA3.1 vector was cotransfected with the FGF11 reporter-luc or TK-luc plasmid into 293T cells, and 48 h later, the cells were lysed with RIPA lysis buffer. The relative luciferase units (RLUs) were calculated by normalizing the FGF11 reporter-luc value to the TK-luc value, and the RLU of the control cells was set to 1. The primers used to construct the MYC-pcDNA3.1 and FGF11 report-luc vectors are listed in **Supplemental Materials: Table S2**.

**Statistical Analysis**

All analyses were performed with SPSS 19.0 and GraphPad Prism 6 software. The numerical data are presented as means ± standard errors of the means (SEM) and were analyzed using one-way analysis of variance (ANOVA) to determine the statistical significance of differences among groups or a standard two-tailed Student’s t-test or paired Student’s t-test to compare differences among subgroups. The nonparametric data were analyzed using Mann–Whitney U tests. Differences were considered statistically significant if P < 0.05. * P < 0.05; ** P < 0.01. Unless indicated otherwise, the experiments were performed without duplicates due to restricted amounts of materials.

**REFERENCES**

1. Li J, Chen Q, He J, Li Z, Tang X, Chen S, et al. Phase I trial of adoptively transferred tumor-infiltrating lymphocyte immunotherapy following concurrent chemoradiotherapy in patients with locoregionally advanced nasopharyngeal carcinoma. *Oncoimmunology* (2015) 4(2):e976507. doi: 10.4161/23723556.2014.976507.

2. Ye SB, Zhang H, Cai TT, Liu YN, Ni JJ, He J, et al. Exosomal miR-24-3p impedes T-cell function by targeting FGF11 and serves as a potential prognostic biomarker for nasopharyngeal carcinoma. *J Pathol* (2016) 240(3):329-40. doi: 10.1002/path.4781.

3. Subramanian A, Tamayo P, Mootha VK, Mukherjee S, Ebert BL, Gillette MA, et al. Gene set enrichment analysis: a knowledge-based approach for interpreting genome-wide expression profiles. *Proc Natl Acad Sci U S A* (2005) 102(43):15545-50. doi: 10.1073/pnas.0506580102.
